# Supplementary material for: Variability of Mitochondrial DNA Heteroplasmy: Association with Asymptomatic Carotid Atherosclerosis
Source: Biomedicines. 2024 Aug 15;12(8):1868. doi: 10.3390/biomedicines12081868 (PMC11351276; doi:10.3390/biomedicines12081868)
Supplement: Supplementary file 1 [file biomedicines-12-01868-s001.zip › Sazonova M A Table S114 08 2024.pdf]

Table S1. Specificity and sensitivity data for 10 investigated mutations

Number	Mutation	Specificity	Sensitivity
1	m.5178C>A	0,621	1,000
2	m.1555A>G	1,000	1,000
3	m.13513G>A	1,000	1,000
4	m.652delG	1,000	1,000
5	m.14846G>A	0,966	1,000
6	m.12315G>A	0,759	1,000
7	m.14459G>A	0,552	1,000
8	m.652insG	0,966	1,000
9	m.3256C>T	1,000	1,000
10	m.15059G>A	0,931	1,000
