# Supplementary material for: Variability of Mitochondrial DNA Heteroplasmy: Association with Asymptomatic Carotid Atherosclerosis
Source: Biomedicines. 2024 Aug 15;12(8):1868. doi: 10.3390/biomedicines12081868 (PMC11351276; doi:10.3390/biomedicines12081868)
Supplement: Supplementary file 1 [file biomedicines-12-01868-s001.zip › Sazonova M A Table S2 14 08 2024.pdf]

Table S2. Specificity and sensitivity data for proatherogenic and antiatherogenic mutations in study participants from the Novosibirsk region

Number	Group of mutations	Specificity	Sensitivity
1	Proatherogenic (m.652delG, m.3256C>T, m.5178C>A and m.12315G>A)	1,000	1,000
2	Antiatherogenic (m.652insG, m.13513G>A and m.14846G>A)	1,000	1,000
