# Supplementary figures and images for: Variability of Mitochondrial DNA Heteroplasmy: Association with Asymptomatic Carotid Atherosclerosis
Source: Biomedicines. 2024 Aug 15;12(8):1868. doi: 10.3390/biomedicines12081868 (PMC11351276; doi:10.3390/biomedicines12081868)

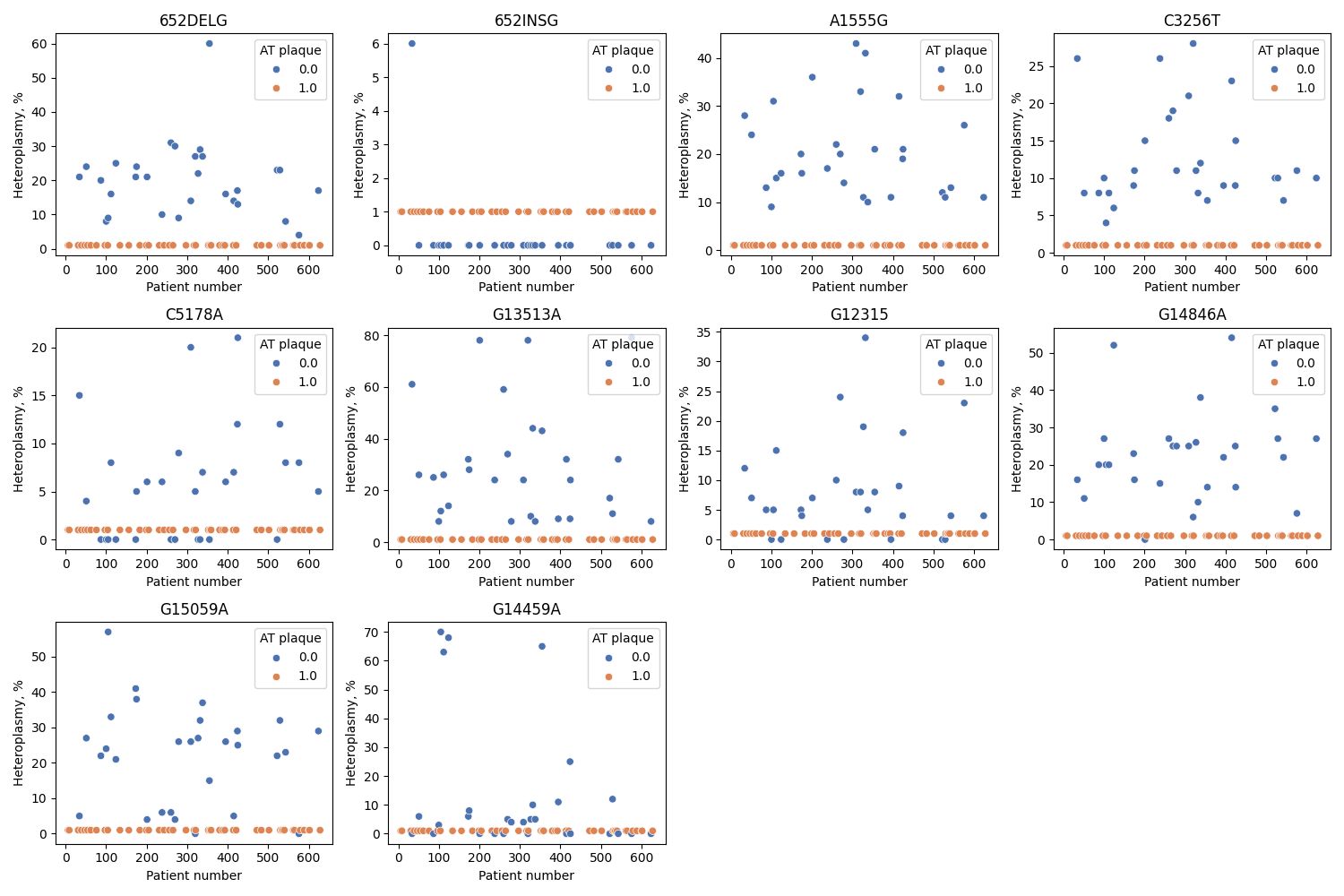

Supplement: Supplementary file 1 [file biomedicines-12-01868-s001.zip › Sazonova M A Figure S1 14 08 2024.jpg]
